# A comparative study of Candida species diversity among patients with oral squamous cell carcinoma and oral potentially malignant disorders

**DOI:** 10.1186/s13104-020-05336-3

**Published:** 2020-10-20

**Authors:** Sankar Leena Sankari, Krishnan Mahalakshmi, Venkatesan Naveen Kumar

**Affiliations:** 1 Department of Oral Pathology and Microbiology, Sree Balaji Dental College and Hospital, Bharath Institute of Higher Education and Research, Chennai, Tamil Nadu 600100 India; 2 Department of Microbiology, Research Lab for Oral-systemic Health, Sree Balaji Dental College and Hospital, Bharath Institute of Higher Education and Research, Chennai, 600100 Tamil Nadu India; 3 Research Lab for Oral-systemic Health, Sree Balaji Dental College and Hospital/ImmuGenix Biosciences Pvt Ltd, No 16/2 Nattal Garden 1st Street, Perambur, Chennai, Tamil Nadu 600011 India

**Keywords:** *Candida*, Oral squamous cell carcinoma, Oral potentially malignant disorder, Polymerase chain reaction, Restriction fragment length polymorphism

## Abstract

**Objectives:**

To determine the prevalence of Candida species by PCR–RFLP method in the saliva of patients with oral squamous cell carcinoma (OSCC), oral potentially malignant disorders (OPMD) and healthy cohorts. Unstimulated saliva was collected from patients with OSCC (n = 97), OPMD (n = 200), and healthy controls (n = 200). Candida species were isolated using the standard protocol. The isolates were identified using phenotypic and genotypic methods. The odds/risk ratio was calculated using Pearson’s Chi-square test. The significance of Candidal carriage was calculated by independent T-test.

**Results:**

Oral Candidal carriage was 72.2%, 58% and 20.5% among patients with OSCC, OPMD, and healthy controls respectively. The oral Candidal carriage in OSCC and OPMD was highly significant (p = 0.0001). Non *albicans Candida* predominated over *Candida albicans*. Candida species were diverse among the study groups with a predominance of *Candida krusei, Candida tropicalis*, and *Pichia anomala* formerly *Candida pelliculosa*. *P. anomala* occurrence outnumbered in health. The odds/risk ratio for OSCC and OPMD were 4.25/11.87 and 3.52/6.99 respectively. A high prevalence of non *albicans Candida* was observed both in all the three groups (OSCC, OPMD and healthy controls). High odds and risk ratio associates Candida species to OSCC and OPMD. *Candida famata* may be associated with OSCC and OPMD.

## Introduction

Globally oral squamous cell carcinoma (OSCC) is the foremost cause for mortality. OSCC is the sixth most common cancer reported globally with an annual incidence of over 300,000 cases. It is the 12th most common cancer in women and sixth in men. About 62% of the cases arise in developing countries. In South-central Asia, it is the third most common type of cancer. In South and South East Asia, high incidence of oral cancer is found in Sri Lanka, India, Pakistan and Taiwan [1]. The age-adjusted rates can vary from over 20 per 100,000 populations in India, to 10 per 100,000 in the United States, and less than 2 per 100,000 in the Middle East [2].

OSCC are most often preceded by clinically apparent oral potentially malignant disorders (OPMD).The presence of Candida species in oral leukoplakia has strengthened its association to with OSCC as well [3]. *Candida* species have been linked to etiopathogenesis of OSCC and oral potentially malignant disorders considering its potential to nitrosylate *N*-benzyl methylamine [4, 5].

*Candida* are oral commensals, wherein it gains a precedence when defence mechanisms are compromised or there is a change in local oral microenvironment that is conducive to *Candida* growth such as low oxygen, low pH and an anaerobic environment leading to a diverse group of infections [3]. Changes in the epithelium with the presence of *Candida* may predispose to Candidal infection which may induce epithelial dysplasia and malignancy [5]. Leukoplakia with Candidal infection has a higher rate of malignant transformation than uninfected leukoplakia [6]. Lesions infected with *Candida* have shown progressively more severe dysplasia compared to patients without Candidal infection [5].

With considerable evidence associating Candida with OSCC & OPMD, the present study aims to assess the prevalence of different *Candida* species among patients with OSCC, OPMD and to compare with normal healthy controls by genotypic methods (PCR–RFLP).

## Main text

### Methods

Study Design: The present study compares the prevalence of Candida species in the saliva of OSCC and OPMD patients with healthy subjects by phenotypic and genotypic methods. The period of recruitment was between December 2014 to June 2019 Study population: The study population comprised of patients with oral squamous cell carcinoma (n = 97), Oral potentially malignant disorders (n = 200) and healthy controls (n = 200). The patients with OSCC & OPMD were recruited from outpatient clinic, Department of Oral Pathology and Microbiology and Centre of Oral Cancer Prevention, Awareness and Research, Sree Balaji Dental College and Hospital. Oral hygiene was assessed using Oral hygiene Index-Simplified (Greene & Vermillion 1964) and categorized as poor, fair, or good [7]. Subjects with poor oral hygiene were not included in the study as poor oral hygiene is considered principal risk factor for candidal colonization. Patients before any form of treatment and willing for biopsy were included for the study group. All the patients in OSCC & OPMD group had habits of smoking/tobacco chewing along with alcohol consumption. Patients with uncontrolled diabetes, immunosuppression, denture wearers, poor oral hygiene status and patients receiving steroid or antibiotic, antifungal therapy for past three months were excluded. Age and gender matched subjects with no history of systemic diseases, oral mucosal lesions, and deleterious habits were recruited for the control group.

### Saliva collection and determination of colony forming units/ml

Unstimulated whole saliva was collected by the “draining” method. The subject’s head was tilted forward so that saliva moved toward the anterior region of the mouth and the pooled saliva (2 ml) was collected into a wide-mouthed sterile container [8]. The sample was then immediately transported to the microbiology laboratory for isolation and identification of *Candida* species. 10 μl of saliva was inoculated onto Sabouraud’s dextrose agar (SDA) plate and incubated at 37 °C for 1 week. The purity was checked by Gram staining. Colony count was performed by a digital colony counter and expressed as colony forming unit (cfu)/ml of saliva.

### Identification of Candida species

#### Phenotypic methods

The identification of *Candida* species was done by standard phenotypic methods (CHROM agar, germ tube tests, chlamydospore formation on cornmeal agar, sugar assimilation, and fermentation tests). Genotypic confirmation was performed by Polymerase chain reaction–restriction fragment length polymorphism (PCR–RFLP) method.

#### Genotypic assay

##### DNA extraction

DNA extraction was performed by boiling lysis method [9]. Single *Candida* colony from a pure fresh SDA plate was picked and inoculated into 200 μl of sterile milli-Q water and kept for 10 min in a heat block (Rivotek, India) at 100 °C. The extracted DNA after incubation at 100 °C was kept in a − 20 °C deep freezer for 10 min and then centrifuged at 10,000 rpm for 5 min. Supernatant was stored at − 20 °C and used as template for PCR assay [10].

#### Polymerase chain reaction

**Fig. 1:**
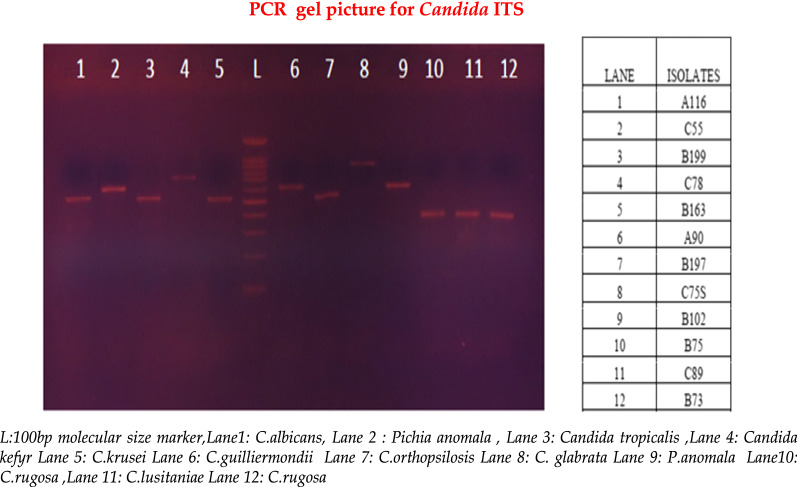
Gel electrophoresis picture showing the PCR amplicons of varied Candida species

PCR was performed for the *Candida* species targeting ITS1-5.8SrDNA-ITS2 region. The PCR mixture (25 µl reaction volume) comprised 10 pM of Candida-ITS-primers ITS1 (5′-TCCGTAGGTGAACCTGCGG-3′) and ITS4(5′-TCCTCCGCTTATTGATATGC-3′) [11], 0.4 mM of dNTP mix, 1 unit of Taq polymerase, 2.5 µl of 10X PCR buffer with MgCl_2_, and 2 µl of DNA template. The PCR reaction was carried out in a Veriti 96 Thermal Cycler (Applied Biosystems, USA). The thermal cycling conditions included an initial denaturation at 94 °C for 3 min followed by 40 cycles at 94 °C for 20 s, 55 °C for 30 s and 72 °C for 45 s, and successively final extension at 72 °C for 5 min. The PCR amplicons were fractionated by gel electrophoresis in 1% agarose with ethidium bromide (0.5 µg/ml) for 25 min at 135 V using Mupid-exU system (Takara, Japan). The gel was examined under BioGlow UV Transilluminators (Crystal Technology, USA) (Fig. 1).

#### Restriction fragment length polymorphism

**Fig. 2:**
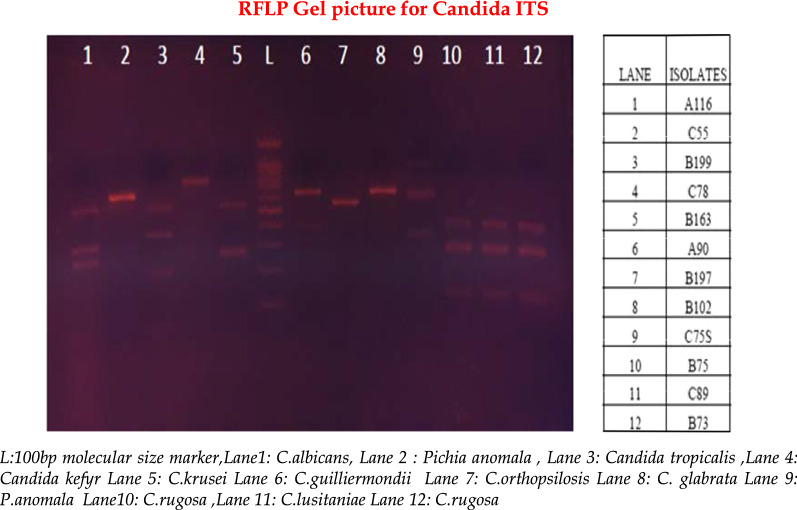
Gel electrophoresis picture showing the PCR fragments of restriction digestion with MspI restriction enzyme

ITS PCR product (8.8 µl) was digested with 0.2 µl MspI (4U) restriction enzyme (New England Biolabs) and 1 µl 10X Enzyme Buffer [11]. The restriction digestion was performed in Veriti 96 Thermal Cycler (Applied Biosystems, USA). The incubation temperature and timing for the mix was 37 °C and 60 min respectively. This was followed by heat inactivation at 85 °C for 5 min. The RFLP products were analysed by gel electrophoresis (2% agarose gel with 0.5 µg/ml ethidium bromide) and the restriction patterns were compared with in silico restriction pattern by pDRAW32 (V 1.1.140) with the sequences from NCBI (Fig. 2).

To authenticate the RFLP based identification of *Candida* species, representative isolates from each species were sequenced using ITS1 primer using ABI PRISM^®^BigDye™ Terminator and ABI 3730XL sequencer (Applied Biosystem, USA) at Macrogen Inc. (Seoul, Korea). Species identification of Candida was achieved by comparing the nucleotide sequence of ITS1-5.8SrDNA-ITS2 region against known sequences available in the GenBank database using the Basic Local Alignment Search Tool (BLAST), (https://blast.ncbi.nlm.nih.gov/Blast.cgi). The *ITS* gene sequences of the isolates was deposited in the NCBI GenBank database and the accession numbers obtained for *C. tropicalis, C.lusitaniae, Pichia anomala formerly C.pelliculosa, Pichia anomala, C. albicans, C.lusitaniae, C.glabrata, C. guilliermondii, C.krusei* and *C. albicans* were *MN394869, MN394870, MN394871, MN39487, MN394872, MN394874, MN394875, MN394876, MN394877* and *MN394878* respectively.

### Statistical analysis

The significance of oral Candidal carriage was calculated using independent T test. Odds and risk ratio of *Candida* to OSCC and OPMD was calculated using Pearson’s Chi square test with a confidence interval of 95%. P value < 0.05 was considered significant.

### Results

Demographic details and prevalence of *Candida* species among the three groups is shown in the Table 1. Predominance of non *albicans Candida* species was observed in all three groups. Among OSCC group *C. krusei* (21%), *C. tropicalis* (21%), *Pichia anomala* (21%)*, C. famata* (17%), *C. glabrata* (5%), *C. rugosa (6%), C. orthopsilosi*s (4%), *C. keyfr* (2%), *C. lusitaniae* (2%) and *C. stellatoides* (1%) were the non *albicans Candida* species isolated. *P. anomala* formerly *C. pelliculosa* (33%), *C. krusei* (27%), *C. tropicalis* (10%), *C. famata* (9%), *C. rugosa* (7%), *C. guilliermondii* (5%), *C. parasilopsis* (3%), *C. orthopsilosis* (3%), *C. glabrata* (1%), *C. stellatoides* (1%), *C. intermedia* (1%) were the non *albicans Candida* species isolated in OPMD group. The non *albicans Candida* species from the healthy controls were *P. anomala* formerly *C. pelliculosa* (40%), *C. tropicalis* (24%), *C. krusei* (17%)*, C. guilliermondii* (4%), *C. parasilopsis* (4%), *C. orthopsilosis* (4%), *C. dubliniensis* (4%), *and C. nivariensis* (4%).

**Table 1:** Demographic details and Prevalence of Candida species in OSCC, OPMD and Control

Groups	Sex n (%)		Total	Age range (years)	Oral Candidal Carriage (%)	*C. albicans* (%)	Non *albicans Candida* (%)	Presence of multiple *Candida* species in the same subject (%)
	Female	Male						
OSCC	19 (19.5)	78 (80.4)	97	31–74	72.2	42.6	72.1	14.7
OPMD	11 (5.5)	189 (94.5)	200	24–76	58	44.8	66.4	7.7
Healthy controls	15 (7.5)	185 (92.5)	200	22–65	20.5	43.5	69.2	15.4

Prevalence of *P. anomala* was high among health (43.5%) followed by OPMD (33%) and OSCC (21%). The odds/risk ratio for OSCC and OPMD were/95% CI 11.87, 6.69–21.05/95% CI 4.25, 3.03–5.96 and 95% CI 6.99, 4.38–11.15/95% CI 3.52, 2.52–4.91 respectively. Odds ratio and Risk ratio for oral candidal carriage among OSCC & OPMD was found to be highly significant (p = .001). The mean cfu/ml was significantly high in OSCC(18601.04, p = 0.000) and OPMD (4238, p = 0.001) compared to healthy controls (222).

### Discussion

The results of the present study show a highly significant oral candidal carriage among OSCC and OPMD groups compared to healthy cohorts. Majority of the earlier studies on oral Candidal carriage in OSCC and OPMD have employed solely phenotypic assays and hence the present study focused to discuss only the literature with similar genotypic methodology. The present study reports a high prevalence of non *albicans Candida* in all the three groups. (Table 1) This finding was not in line with a similar study by Alnuaimi et al. and Shokohi et al. [12, 13]. The non *albicans Candida* species diversity was high in OSCC and OPMD compared to healthy controls. Here, we report high species diversity in OSCC and OPMD. This may be attributed to the analysis by in silico restriction pattern by pDRAW32. Few subjects in all the three groups presented mixed colonization of *Candida* species in their saliva (Table 1). Interestingly, the present study by culture method identified 10 different non *albicans Candida* species among OSCC compared to an oral mycobiome study who have reported only three different *Candida* species by performing Next Generation Sequencing coupled with a species-level taxonomy assignment algorithm [14].

Among the non *albicans Candida* all the three groups showed a high prevalence of *C. krusei, C. tropicalis* and *Pichia anomala* formerly *Candida pelliculosa* while a similar Iranian study by Shokohi et al. reported predominance of *C. glabrata*, *C. tropicalis* and *C. krusei* in patients with OSCC [13]. To the best of our knowledge this may be the first study to report the presence of *P. anomala* formerly *C. pelliculosa* in all the three groups. *P. anomala* oral carriage was found to decrease in OSCC and OPMD conditions compared to health. This finding signifies the role of *P. anamola* in health. This is in coherence with a study on HIV patients who has reported the antagonistic effect of *P. anamola* on *Candida* species [15]. *Candida famata* was totally absent in the saliva of healthy controls. Conversely *C. famata* was present in the saliva of OSCC (17%) and OPMD (9%) patients. The present study detected *C*. *parapsilosis*, *C*. *guilliermondii* and *C*. *dubliniensis* whereas a similar study by Alnuaimi et al. did not report these *Candida* species [12]. Diverse candida species in the present study may be attributed to PCR–RFLP method of identification.

High odds and risk ratio for oral Candidal carriage among OSCC & OPMD suggests their association to these conditions. The odds of oral Candidal carriage among OSCC in the present study are 4.25. This finding suggests *Candida* infection as a risk factor for OSCC which is in concurrence with a previous study [16]. Non *albicans Candida* was the predominant isolate in OPMD which is in contrast to several other studies who have followed phenotypic methods alone for identification of *Candida* species [17, 18]. In OPMD, erythroplakia and erythroleukoplakia which has high malignant transformation potential presented with high oral Candidal carriage. The Candidal carriage in OSMF was higher than leukoplakia. *C. albicans* was the predominant (87.5%) species isolated in OSMF patients. Majority of the OSCC patients had documented risk factors for OSCC such as the habit of alcohol consumption, smoking and chewing of tobacco products which are to be considered for their confounding effects. However, these risk factors may have attributed in part for the high incidence of oral Candidal carriage among OSCC patients. Yet there were sizable number (13.4%) of subjects in OSCC group who did not have any of the deleterious habits. High Oral Candidal carriage in OSCC and OPMD may be an added burden in these patients which adds on to their morbidity.

High prevalence of oral candidal carriage in patients with OSCC and OPMD suggests their association to these conditions. This finding recommends the need for their suppression to prevent opportunistic infections which may help in subsequent prevention of transformation of OPMD to OSCC. In silico restriction pattern has helped in elucidating the Candidal species diversity in OSCC & OPMD. Non *albicans Candida* was the predominant group supporting its emergence in recent times. This may be the first study to report significant presence of *P. anomala* in all the three groups. The findings of the present study with high Candida diversity may be attributed to the genotypic method of identification in particular, PCR–RFLP method.

## Limitations

- Samples from different geographical regions would enhance significance to the study.
- The association of Candida species to OSCC and OPMD can be further strengthened if significant number of patients with absence of deleterious habits were included.

## Data Availability

All data generated or analysed during this study are included in this published article [and its supplementary information files].

## Abbreviations

OSCC: Oral squamous cell carcinoma
OPMD: Oral potentially malignant disorders
PCR: Polymerase chain reaction
RFLP: Restriction fragment length polymorphism
